# HybridCATNet: an explainable CNN–Transformer framework with early multi-channel fusion and uncertainty estimation for pneumonia detection from chest X-rays

**DOI:** 10.3389/fdgth.2026.1769444

**Published:** 2026-07-22

**Authors:** Abdullah, Muhammad Ateeb Ather, Kinza Sardar, Zulaikha Fatima, José Luis Oropeza Rodríguez, Carlos Guzmán Sánchez-Mejorada, Rolando Quintero Téllez, Miguel Jesús Torres Ruiz

**Affiliations:** 1 Centro de Investigación en Computación (CIC), Instituto Politécnico Nacional (IPN), Mexico City, Mexico; 2 Department of Computer Sciences, Bahria University, Lahore, Pakistan; 3 Department of Software Engineering, University of Management and Technology, Lahore, Pakistan; 4 Faculty of Allied Health Sciences, Superior University, Lahore Campus, Lahore, Pakistan

**Keywords:** attention mechanism, Bayesian dropout, chest X-ray, explainable AI, hybrid CNN–Transformer, multi-modal fusion, pneumonia detection, uncertainty estimation

## Abstract

**Introduction:**

Pneumonia remains a leading cause of morbidity and mortality worldwide, particularly among children and older adults, making rapid and reliable interpretation of chest X-rays essential for timely clinical decision-making. This study proposes HybridCATNet, an explainable and uncertainty-aware deep learning framework for automated pneumonia detection from chest radiographs.

**Methods:**

HybridCATNet integrates a convolutional neural network and a vision transformer to capture both fine-grained local radiographic features and long-range anatomical dependencies. To incorporate clinically relevant image priors, a nine-channel early multi-channel fusion strategy combines raw grayscale chest radiographs, lung-segmented masks, and edge-enhanced structural maps. A Cascaded Spatial–Channel Attention Module further refines diagnostic feature representation, while Bayesian dropout is employed during inference to estimate predictive uncertainty. The model was trained and evaluated on a balanced multi-institutional dataset comprising 8,478 authentic chest radiographs without synthetic oversampling using 10-fold cross-validation. Generalizability was further assessed on two independent external validation cohorts. Explainability was evaluated using Grad-CAM and SHAP.

**Results:**

HybridCATNet achieved an area under the receiver operating characteristic curve (ROC-AUC) of 0.9953, an accuracy of 96.93%, a sensitivity of 98.13%, and a specificity of 95.72% in 10-fold cross-validation. External validation demonstrated robust cross-domain performance, with an accuracy of at least 94.8% across both independent cohorts. Explainability analysis showed strong agreement between model-generated attention maps and radiologist-annotated pathological regions, achieving an intersection-over-union of 0.812 and a faithfulness correlation of 0.921.

**Discussion:**

The results demonstrate that HybridCATNet provides accurate, interpretable, and uncertainty-aware pneumonia detection while maintaining strong generalization across external datasets. By integrating multi-channel image fusion, hybrid CNN–Transformer feature learning, attention-guided refinement, predictive uncertainty estimation, and explainability, the proposed framework offers a clinically transparent decision-support approach with potential to assist pneumonia screening and improve confidence in AI-assisted radiographic diagnosis.

## 1 Introduction

Pneumonia remains one of the most frequent causes of morbidity and mortality worldwide, with a disproportionately high burden among pediatric and geriatric populations. This long-term liability places significant strain on healthcare systems in both low-resource and high-resource settings. The most common type of imaging that is used to screen and diagnose pneumonia is chest radiography (CXR) due to its cost-effectiveness, quick acquisition, and universal availability (1). However, the accuracy of CXR interpretation requires considerable clinical astuteness and is prone to inter-rater variation, especially in instances of subtle or nascent disease when radiographic opacities can be diffused or weak (2). These limitations have increased international efforts to create automated, reliable, and explainable artificial intelligence (AI) systems to assist radiologists and other frontline clinicians in detecting pneumonia (3, 4).

Early pneumonia classifiers that used deep learning utilized, mainly, traditional convolutional neural networks (CNNs) such as VGG, ResNet, and DenseNet (5). These models performed reasonably well because they used strong inductive biases on spatial locality and translation invariance. In recent years, Vision Transformers (ViTs) and hybrid CNN–Transformer architectures have become promising alternatives, making use of long-range dependency modeling and global contextual reasoning (6–8). Despite the success of these transformer-based models in the field of natural image analysis, they are problematic to apply to the field of medical imaging because of small datasets, cross-institutional changes in domain, and the risk of learning spurious correlations (9, 10). At the clinical level, the appearance of pneumonia is often subtle, with texture-dependent opacities, the detection of which depends not only on the fine-grained local characteristics but also on the anatomically consistent global context—an interaction that cannot be fully encoded in CNNs (or Transformers alone) (11).

At the same time, training data integrity has become a serious bottleneck in medical AI translation (12). Many studies have extensively used synthetic oversampling, aggressive augmentation, or adversarial data generation to combat extreme class imbalance (13). Although these methods can increase apparent accuracy, they risk distorting natural clinical distributions and decreasing generalizability. In addition, existing models typically work with single-channel grayscale CXRs, thus implicitly assuming that raw pixel intensity provides information on the anatomical boundaries, texture gradients, and structural priors (14, 15). This limitation results in imperfect use of model capacity. It has been shown in recent times that the explicit combination of anatomical and structural priors—such as lung segmentation masks and edge-enhanced representation—can significantly improve robustness and interpretability. However, systematic integration of such modalities in a coherent, end-to-end system has not been well studied.

In order to address these limitations, the present study introduces HybridCATNet, a clinically oriented hybrid deep learning network developed for the automated identification of pneumonia based on chest X-ray images. It is designed with four salient advancements, as mentioned in the proposed methodology.

The main contributions of this work are summarized as follows:

1. We propose HybridCATNet as a hybrid CNN–Transformer architecture for binary pneumonia detection that combines convolutional local lesion encoding with transformer-based global anatomical-context modeling in a single end-to-end framework.
2. We develop a nine-channel early-fusion strategy that concatenates replicated raw grayscale radiographs, lung-masked radiographs, and edge-enhanced maps at the input level, followed by a Cascaded Spatial–Channel Attention Module (CSCAM) to emphasize diagnostically relevant pulmonary regions and suppress non-lung or device-related activations.
3. We establish a balanced training paradigm using authentic radiographs, rather than synthetic oversampling, and demonstrate its effectiveness in improving convergence stability, sensitivity, calibration reliability, and feature interpretability under clinically realistic data distributions.
4. We rigorously evaluate model reliability and transparency, integrating Bayesian uncertainty quantification for risk-aware inference and validating interpretability through Gradient-weighted Class Activation Mapping (Grad-CAM) and Shapley Additive Explanation (SHAP) analyses, alongside extensive cross-domain and robustness testing to confirm generalization across heterogeneous imaging conditions.

Together, these contributions position HybridCATNet as a scalable, interpretable, and uncertainty-aware diagnostic framework that bridges the gap between high-performance deep learning models and the practical requirements of real-world radiological deployment.

The primary objective of the research is to design and rigorously test a powerful, understandable, and generalizable AI-based diagnostic system for pneumonia detection that creates a good balance between diagnostic granularity and computational efficiency. This work aims to illustrate that, through extensive cross-validation, cross-domain testing, ablation studies, and extensive explainability audits, it is possible to achieve reliable performance in clinical applications without the use of synthetic data, while maintaining transparent and unbiasedness. HybridCATNet brings anatomical, structural, and contextual logic together within a single framework, which represents state-of-the-art advancement in medical image analysis. It provides a concrete way toward reliable AI implementation in clinical radiological processes.

## 2 Related work

The state of automated pneumonia diagnosis based on CXR has experienced rapid development during 2024–2025, shifting the state of monolithic deep learning models to clinically grounded, data-sparse, and anatomy-constrained learning models. Recent studies can be grouped into four interconnected topics, namely, efficient CNN-centric learning, multi-modal combination of anatomical and structural features, advanced attention mechanisms, and natural dataset harmonization strategies. These developments are presented in a chronological and conceptual order in the following review with the aim of contextualizing the HybridCATNet framework.

### 2.1 Efficient CNN-based methods for pneumonia detection

Efficient CNN-based methods have been widely investigated for pneumonia detection. A descriptive comparative study of modern CNN models for detecting pneumonia in children found that EfficientNetV2L achieved better results in capturing diffuse and low-contrast opacities compared with more complex residual and inception-based models (16). These findings challenged the assumption that deeper architectures always provide better clinical performance and highlighted the importance of compound scaling and progressive learning in medical imaging. Expanding on this direction, the DenseNet family was evaluated, showing diminishing returns after DenseNet169, where additional depth provided limited improvement in accuracy while increasing overfitting risk and computational load (17). This trend was further supported by work combining EfficientNet with Squeeze-and-Excitation blocks, which improved convergence stability and generalization compared with transformer-based backbones on small-to-medium-sized medical datasets (18). In contrast, pure Vision Transformer (ViT) models were explored for multi-class pneumonia classification, achieving good predictive performance but also showing high vulnerability to negative transfer because of their strong dependence on large-scale pre-training, limiting their applicability in low-data clinical settings (19).

### 2.2 Multi-modal anatomical and structural priors

The introduction of explicit anatomical and structural priors has emerged as an important research direction. The Segmentation-Assisted Fusion-Based Classification framework combined lung segmentation masks, obtained through a partial convolutional segmentation network, with original CXR images to reduce non-diagnostic background artifacts (20). Although effective, this strategy mainly relied on late or middle fusion. Another study incorporated handcrafted structural priors by integrating classical edge detection methods, including Sobel and Canny, with deep classifiers, improving discrimination between hazy pathological boundaries and normal tissue gradients (21). Multi-modal learning was further advanced through the CaMcheX framework, which combines visual radiological data with auxiliary clinical text priors and improves performance in scenarios with rich metadata (22). MobileNetV2-based deep features and Sobel-based edges have also been combined with a support vector machine (SVM) classifier; however, the non-end-to-end nature of this approach limited complete gradient optimization (23). A detection-transformer-based model, CD-DETR, was proposed for localization-oriented pneumonia detection using multi-scale feature fusion, with attention focused on bounding boxes rather than whole-image classification (24).

### 2.3 Attention mechanisms for feature refinement

Attention mechanisms have also been extensively refined for pneumonia detection. The Convolutional Block Attention Module was integrated with VGG16, applying channel and spatial attention sequentially to improve feature discrimination in pneumonia classification (25). The Fuzzy Channel Selective Spatial Attention Module (FCSSAM) used fuzzy logic to dynamically weight channel-wise importance and reduce the rigidity of sequential attention pipelines (26). A hybrid deep attention framework combining VGG16 and VGG19 with deeper multi-stage attention further showed that single-pass attention may not fully capture the complexity of medical images with lesions of varying sizes (27). MedViT V2 introduced Dilated Neighborhood Attention to efficiently model multi-scale context without incurring the high computational cost of global self-attention (28). A Dual-Branch Attention Fusion Network handled features from two attention branches in parallel before merging them, thereby preserving representational diversity (29). Similarly, ensemble-like feature-preserving reweighting approaches showed strong performance when attention mechanisms were designed to retain, rather than compress, feature diversity (30).

### 2.4 Dataset harmonization and class balancing

Recent studies have increasingly emphasized the importance of dataset integrity and realistic class balancing. Comparisons between synthetic oversampling and GAN-based augmentation methods have shown that excessive synthetic data may introduce feature hallucination and reduce generalization (31). The CoMViT framework demonstrated significant performance degradation during cross-domain testing when models were trained using heavily synthetically balanced data, indicating that artificially distorted distributions can make models fragile (32). As a result, the research community has shifted toward real data harmonization strategies. The RSNA Pneumonia Detection Challenge dataset has become one of the most commonly used datasets for evaluating normal and pathological clinical distributions and assessing cross-institutional robustness (33). Domain generalization principles in medical imaging further highlight the value of diverse real-world data for learning diagnostic features that remain invariant across settings (34). Moreover, models trained on realistically balanced, multi-source data have been shown to perform better under domain shifts, supporting the argument that clinical AI systems should be developed using realistic data distributions (35).

DenseNet-based architectures remain highly competitive for chest X-ray analysis due to their dense feature reuse, improved gradient propagation, and parameter efficiency (36). The effectiveness of DenseNet-based architectures in thoracic disease detection was prominently demonstrated through DenseNet-121, which became a strong benchmark for pneumonia classification (37). More recent comparative investigations have further confirmed that DenseNet and EfficientNet variants achieve robust baseline performance when trained using rigorous preprocessing, transfer learning, and patient-wise partitioning protocols (38, 39).

### 2.5 Applications of deep learning and machine learning in disease diagnosis, prediction, and clinical decision support

Beyond pneumonia diagnosis, deep learning and machine learning have been increasingly applied to disease diagnosis, prediction, screening, rehabilitation, and clinical decision support systems. These studies are relevant to the present work because they demonstrate the growing importance of hybrid architectures, explainable learning, multi-modal integration, and deployable medical AI in complex healthcare environments. For traumatic brain injury structure detection, a CNN–ViT framework with advanced wavelet transformation fusion demonstrated the benefit of combining convolutional local feature extraction with transformer-based global spatial modeling (40). In biomedical tabular analysis, interpretable multi-dataset learning for breast cancer prediction emphasized cross-dataset generalization and clinically transparent decision-making (41), while broader breast cancer diagnosis research further highlighted the role of deep learning and explainability across imaging modalities (42).

Hybrid and adaptive intelligence has also been applied to wider healthcare domains. A reinforcement learning framework for complex health cognitive systems demonstrated the potential of adaptive models for autonomous clinical decision support (43). In diabetic foot ulcer classification, an explainable and clinically audited hybrid self-supervised framework highlighted the need for interpretability, clinical validation, and mobile deployment (44). Similarly, a context-aware SARS disorder management framework integrated machine learning, multi-agent simulation, and biosensor data for sensor-driven healthcare intelligence (45). In gastrointestinal disease classification, an attention-enhanced ViT-HLNN hybrid ensemble demonstrated the effectiveness of attention-guided hybrid architectures for medical image interpretation (46). The RhythmX^™^ framework further showed the value of interpretable self-supervised contrastive learning for heartbeat classification (47). Collectively, these studies show that medical AI is moving toward hybrid, interpretable, data-efficient, deployable, and patient-centered systems. These trends are particularly relevant to CXR-based pneumonia detection, where local lung textures, global anatomical context, uncertainty awareness, and explainability are essential for reliable automated classification.

### 2.6 Gaps and contribution

HybridCATNet extends beyond these conventional CNN pipelines in two distinct ways. First, it integrates explicit anatomical and structural priors, lung segmentation masks, and edge-enhanced representations through a structured nine-channel multi-modal input that encodes complementary spatial, textural, and morphological information before deep feature extraction. Second, it incorporates a hybrid CNN–Transformer fusion combined with a CSCAM, enabling enhanced global contextual modeling, feature recalibration, and improved calibration stability under cross-domain and heterogeneous institutional settings. This design addresses the limitations of purely convolutional backbones, particularly in scenarios involving domain shift and structural variability in chest radiographs. Taken together, these studies represent a trend toward efficient CNN backbones, anatomically motivated multi-modal inputs (48–50), cascaded and feature-conserving attention processes, and authentic dataset harmonization principles, which are directly applied and inspired by the design of the proposed HybridCATNet framework.

## 3 Methodology

The present research outlines a thorough deep learning architecture for automated pneumonia detection from the chest radiographs, incorporating dataset harmonization, a more sophisticated design of hybrid architecture, and stringent evaluation practices. A balanced corpus was compiled by combining the pediatric chest X-ray dataset with real normal radiographs from the RSNA Pneumonia Detection Challenge, achieving a balanced representation of the classes, radiographic realism, and inter-institutional diversity. Extensive preprocessing, such as deduplication, leakage removal, quality filtering, and multi-modal enhancement, produced a nine-channel composite representation, conveying structural, textural, and anatomical information. The proposed HybridCATNet model incorporates both convolutional and transformer units into a single network, which uses early fusion and cascaded spatial–existing channel attention to learn not only local lesions but also the overall pulmonary environment. AdamW was applied to the network with cosine annealing and focal loss to optimize it, and its performance was tested using the extensive 10-fold cross-validation. Quantitative metrics and interpretability analyses (Grad-CAM, SHAP) demonstrated higher diagnostic accuracy, stability, and fairness compared with current CNN or transformer baselines. The resultant system represents a clinically based, reproducible, and explainable pneumonia detection method and has helped advance the credibility of AI-assisted radiographic diagnosis.

### 3.1 Dataset and preprocessing

**Figure 1:**
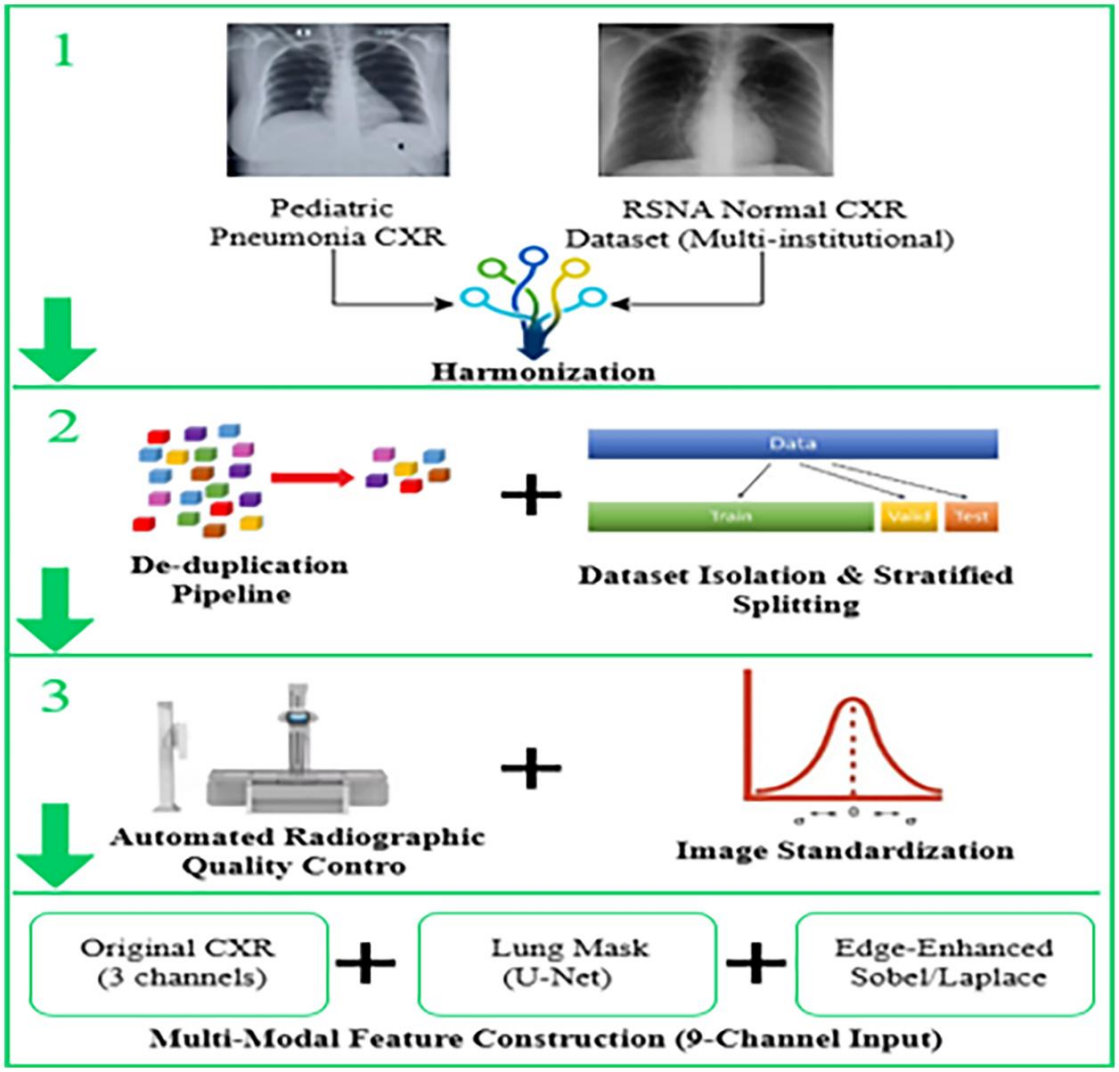
Multi-model feature construction.

The pneumonia detection system was trained on a larger and harmonized corpus of CXR (36) comprising both the original pediatric dataset of 5,856 images and additional normal radiographs obtained through the RSNA Pneumonia Detection Challenge. Integration of RSNA normal cases (37) was done to reduce the imbalance of classes while maintaining radiographic realism, inter-patient variability, and institutional diversity, as shown in Figure 1.

The original dataset consisted of 1,583 normal (27%) and 4,273 pneumonia (73%) CXRs. In order to have a balanced representation, the real normal radiographs were imported into the RSNA Challenge dataset, which had de-identified, high-quality frontal-view CXRs of several different institutions. The resulting dataset included 4,205 normal (49.6%) and 4,273 (50.4%) pneumonia cases, amounting to 8,478 images.

The combination of datasets from heterogeneous sources required systematic harmonization in order to guarantee compatibility, avoid duplication, and eliminate possible data leakage. All images were down-sampled to 224 × 224 and converted into one-channel grayscale to standardize the spatial and intensity size. Histogram equalization and z-score normalization were used to address the contrast differences between datasets, decreasing scanner-specific brightness and exposure variation while maintaining the structural detail.

A two-level duplicate detection pipeline was utilized. First, perceptual hashing (pHash) filtering was used to find visually similar CXRs with a Hamming distance of 5 or less to flag duplicates. Second, ResNet-18 embedding similarity analysis was performed on feature vectors extracted from the images; pairs with cosine similarity >0.98 were manually reviewed and near-duplicates (62 images, 0.7%) were eliminated,ensuring dataset uniqueness and representativenes.

Two complementary evaluation settings were used. In the fixed 70/15/15 hold-out split shown in Table 1, RSNA normal radiographs were used only to balance the training and validation subsets, whereas the fixed hold-out test subset contained only original, unseen pediatric CXR cases. This source-isolated hold-out setting was used for initial sanity checking and baseline evaluation. In contrast, the primary performance estimate was obtained using 10-fold patient-wise stratified cross-validation as illustrated in Figure 4 on the full harmonized dataset of 8,478 images. In this cross-validation setting, RSNA images were not excluded from evaluation folds; instead, all images were kept patient-isolated wherever identifiers were available, duplicate-filtered before folding, and prevented from appearing across training and evaluation folds within the same split. Therefore, RSNA exclusion applies only to the fixed hold-out test setting, while the 10-fold cross-validation results reflect performance across the entire harmonized dataset.

**Table 1:** Final data partitioning.

Data split	Number of images	Percentage	Purpose/description
Training	5,934	70	Used for model learning with balanced, stratified sampling
Validation	1,272	15	Used for model selection, hyperparameter tuning, and early stopping
Test	1,272	15	Used only for final evaluation; contains original, unseen CXR cases not used during training or tuning
Total	8,478	100	Complete harmonized dataset used in the study

Automatic quality filtering was also applied to remove images with motion blur, severe exposure artifacts, or incorrect radiographic projections. This filtering was performed using a CNN-based radiographic quality control network trained on 10,000 manually labelled CXRs. Approximately 3.2% of imported RSNA images were removed during this step before final dataset partitioning.

The following sequence of preprocessing was performed on each CXR to ensure optimal model learning:

(a) Resizing: All images were normalized to 224 × 224 pixels, ensuring uniform input size.
(b) Normalization: Pixel values were normalized to the [0, 1] range, which stabilized gradient updates.
(c) Contrast amplification: Histogram equalization was used to improve the visualization of pulmonary structures.
(d) Lung segmentation: A trained U-Net generated lung masks, which isolated anatomically relevant regions.
(e) Edge enhancement: Sobel filtering emphasized structural and parenchymal boundaries.
(f) Multi-modal input construction: Original grayscale, segmented mask, and edge-enhanced channels were combined to create a nine-channel composite input (three original, three mask, and three edge).

The combination of these networks enabled the integration of textural, morphological, and anatomical responses. By incorporating authentic normal radiographs from an independent dataset rather than relying solely on synthetic augmentation, the pipeline achieved enhanced radiographic realism and morphological diversity, reduced domain and class bias, and improved statistical fairness and generalization robustness. Harmonization measures, de-duplication, and leakage control procedures guarantee data integrity with diagnostic validity and scientific reproducibility, thus providing a rigorous foundation for subsequent model training and cross-validation.

In addition to stratified randomization, patient-level isolation was strictly enforced using available patient identifiers in the metadata of both datasets. All images belonging to a single patient were assigned exclusively to one subset (training, validation, or test), ensuring that no patient appeared across multiple partitions. This precaution prevented information leakage arising from correlated radiographs of the same subject and guaranteed unbiased generalization evaluation.

The lung segmentation U-Net was pretrained independently, and its weights were frozen during classifier training. The segmentation network was not optimized on validation or test images and was not jointly trained with the pneumonia classifier. This separation ensured that no task-specific information from the evaluation subsets influenced feature extraction, thereby eliminating segmentation-induced data leakage.

To prevent data leakage, duplicate detection was completed before any partitioning or cross-validation. Patient-level isolation was enforced wherever patient identifiers were available, ensuring that images from the same patient did not appear in more than one subset or fold. Data augmentation was applied only to the training subset within each experiment and never to validation, hold-out test, or cross-validation evaluation folds. The lung segmentation U-Net was pretrained independently, frozen during classifier training, and not optimized using validation or test images. In the fixed 70/15/15 hold-out setting, RSNA images were excluded from the hold-out test subset. In the 10-fold cross-validation setting, the full harmonized dataset was used, but each fold remained patient-wise isolated and duplicate-free.

This institutional separation created a controlled domain shift evaluation setting in which the model was trained on multi-source data but tested on a distinct clinical distribution. Such cross-source isolation reduced the risk of memorization and better reflected real-world deployment conditions, where models encounter unseen institutional variability. All preprocessing steps, partition indices (seed = 42), and model hyperparameters were fully documented to ensure reproducibility. The dataset composition and filtering criteria were described in sufficient detail to allow independent reconstruction of the experimental setup using the publicly available source datasets.

### 3.2 Model architecture

**Figure 2:**
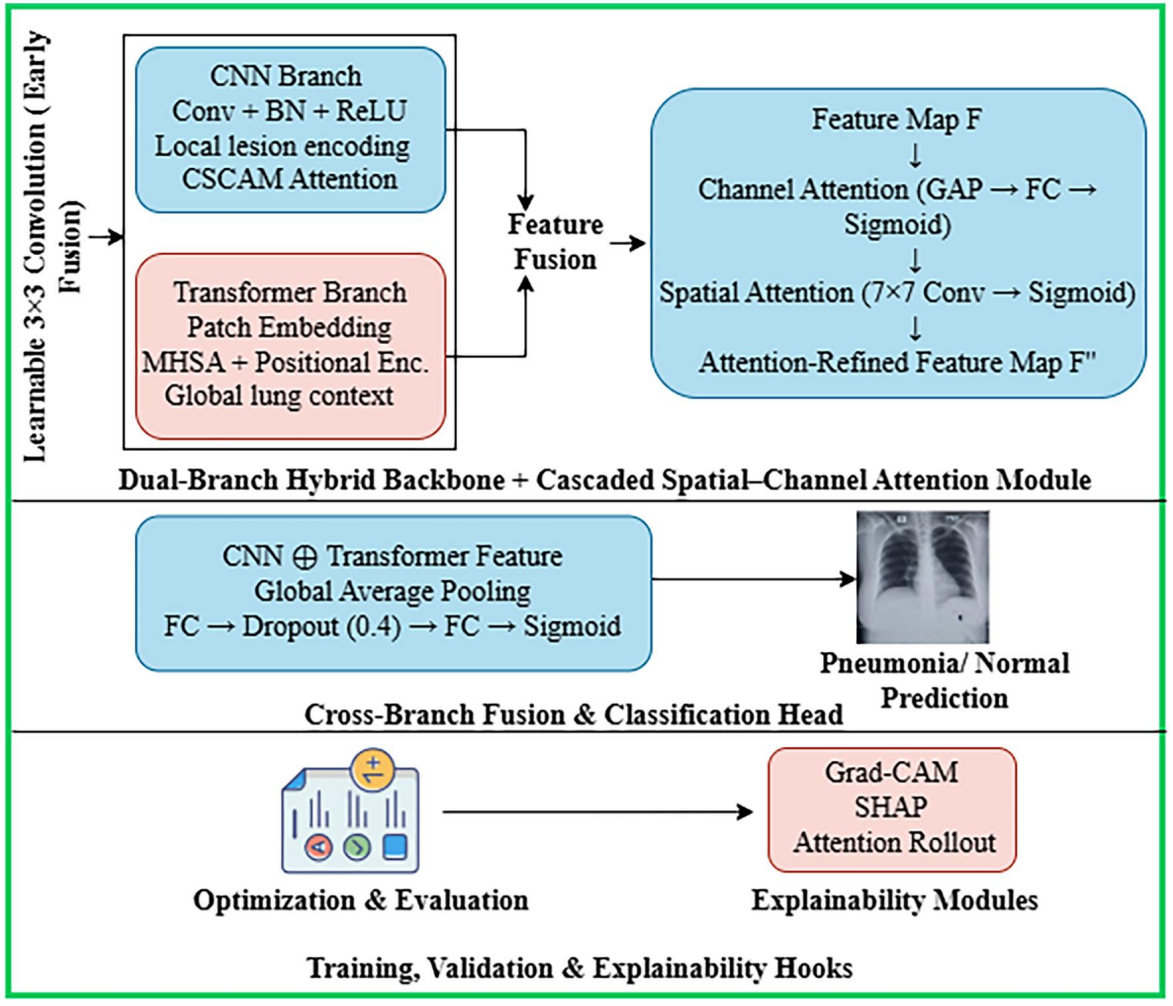
Model architecture.

The proposed HybridCATNet presents a mathematically founded hybrid design that combines convolutional and transformer-based representations in order to achieve high-quality detection of pneumonia from chest radiographs. Its architecture aims to fuse local convolutional priors based on local texture sensitivity and global self-attention dependency based on self-attention in a coherent, end-to-end trainable system, as shown in Figure 2.

The model processes a nine-channel composite input tensor,

$$X\in R^{9\times H\times W},$$

where each triplet of channels corresponds to

(a) original grayscale radiographs ($X_{orig}$),
(b) lung-segmented anatomical priors (U-Net mask  ×  original), represented by ($X_{mask}|$), and
(c) edge-enhanced derivatives via Sobel and Laplacian filters ($X_{edge}$).

Thus, as shown in Equation 1,

$$X=[X_{orig}\;∥X_{mask}\;∥\;X_{edge}]$$ (1)

where “ ∣∣ ” denotes the channel-wise concatenation.

This composite representation is able to capture low-level structure gradients and high-level semantics patterns so that the network can detect opacities and consolidation boundaries.

The nine-channel input is formed by concatenating three complementary representations: raw grayscale radiographs (3 channels from replicated original), edge-enhanced structural maps (3 channels from Sobel and Laplacian filtering), and where each triplet of channels corresponds to: (1) original grayscale radiographs ($X_{orig}$), (2) lung-segmented anatomical priors via U-Net masking ($X_{mask}$), and (3) edge-enhanced structural derivatives via Sobel and Laplacian filtering ($X_{edge}$). This early concatenation strategy enables the network to jointly learn cross-modal interactions from the very first convolutional layer, aligning texture, boundary, and anatomical information before any high-level abstraction takes place, which we empirically found to improve calibration and reduce domain shift sensitivity compared to late fusion.

#### 3.2.1 Hybrid CNN–Transformer backbone

The dual-branch architecture of HybridCATNet comprises a CNN stream for local encoding and a Transformer stream for long-range dependency modeling.

(a) CNN branch: local encoding

The CNN pathway processes the input *X* through sequential convolutional blocks, as shown in Equation 2:

$$F_{cnn}=f_{cnn}(X)=\sigma (\mathrm{BN}(W_{c}*X+b_{c}))$$ (2)

where “ ∗ ” denotes the convolution, $W_{c}$ is the learnable convolution kernel, BN(⋅) represents the batch normalization, and σ(⋅) is the ReLU activation function. This stream captures fine-grained lesion contours, texture irregularities, and boundary sharpness critical for pneumonia localization.

(b) Transformer branch: global context modeling

In parallel, the Transformer branch reshapes the input into a patch-embedding sequence, as shown in Equation 3:

$$Z_{0}=[x_{1}*E;|\;\;x_{2}*E;|\;\;\ldots ;\;\;|\;\;x_{N}*E]+E_{\;pos}$$ (3)

where E is the patch-embedding projection and $E_{\;pos}\;$ denotes positional encoding. Each Transformer layer performs multi-head self-attention (MHSA), as shown in Equation 4:

$$\mathrm{MHSA}(Q,K,V)=\mathrm{Concat}(h_{1},\ldots ,h_{M})*W_{O}$$ (4)

with each head defined as, as shown in Equation 5:

$$h_{i}=\mathrm{softmax}\left(\frac{Q_{i}*K_{i}^{T}}{\sqrt{d_{k}}}\right)*V_{i}$$ (5)

where *Q*, *K*, and *V* are the query, key, and value matrices, respectively, and $d_{k}$ is the dimensionality of each attention head. This mechanism captures inter-lobar correlations, bilateral opacity symmetry, and global radiographic dependencies that traditional CNNs alone may fail to model.

#### 3.2.2 Early fusion and nine-channel integration

Unlike conventional late-fusion strategies, HybridCATNet employs early fusion through a learnable convolutional integration, as shown in Equation 6:

$$F_{0}=\sigma (W_{f}*X+b_{f})$$ (6)

where *W_f_* denotes the learnable fusion kernel, defined as shown in Equation 7:

$$W_{f}\in R^{C\times 9\times 3\times 3}$$ (7)

The early-fusion strategy enables the network to learn cross-modal dependencies between structural and textural representations from the initial layers, reducing feature redundancy and improving training convergence. The early fusion layer acts as a low-level manifold aligner, ensuring that all modalities share a common feature-embedding space and only acquire higher-level abstraction.

#### 3.2.3 Cascaded Spatial–Channel Attention Module

To selectively emphasize diagnostically relevant cues, a CSCAM is integrated within the CNN branch.

(a) Channel attention

Given an intermediate feature map, as shown in Equation 8:

$$F\in R^{C\times H\times W}$$ (8)

Global average pooling is applied to compute the per-channel descriptor, as shown in Equation 9:

$$z_{c}=\frac{1}{H\times W}\overset{H}{\underset{i=1}{\sum }}\overset{W}{\underset{\;j=1}{\sum }}F_{c}(i,j)$$ (9)

The pooled descriptor is then passed through two fully connected layers, as shown in Equation 10:

$$s_{c}=\sigma (W_{2}\;\delta (W_{1}\;z_{c}))$$ (10)

producing per-channel attention weights $s_{c}$. The refined map is obtained as shown in Equation 11:

$$F^{\prime }=s_{c}⊙F$$ (11)

where ⊙ denotes the element-wise multiplication.

(b) Spatial attention

A convolutional projection is then applied, as shown in Equation 12:

$$M_{s}=\sigma (\;f_{7\times 7}(\left[F_{\mathrm{avg}}^{{}^{\prime }};F_{\max}^{{}^{\prime }}\right]))\;$$ (12)

The final attention-weighted feature map is obtained as shown in Equation 13:

$$F^{\prime \prime }=M_{s}⊙F^{\prime }$$ (13)

While suppressing unimportant background patterns like ribs or device shadows, this cascaded sequence (channel → spatial) dynamically re-weights important pulmonary regions, improving perihilar consolidations, basal infiltrations, and diffuse opacity fields.

#### 3.2.4 Cross-branch fusion and classification head

Outputs from both branches are merged, as shown in Equation 14:

$$F_{\mathrm{hyb}}=\mathrm{Concat}(F_{\mathrm{cnn}},F_{\mathrm{trans}})\in R^{D\times H^{\prime }\times W^{\prime }}$$ (14)

This is followed by global average pooling (GAP), two fully connected (FC) layers with dropout (p=0.4), and a sigmoid activation for binary classification, as shown in Equation 15:

$$\hat{y}=\sigma (W_{\mathrm{fc}2}*\delta (W_{\mathrm{fc}1}*\mathrm{GAP}(F_{\mathrm{hyb}})))$$ (15)

The network is trained by minimizing the binary cross-entropy loss, as shown in Equation 16:

$$L=-\frac{1}{N}\overset{N}{\underset{i=1}{\sum }}[y_{i}\log({\hat{y}}_{i})+(1-y_{i})\log(1-{\hat{y}}_{i})]$$ (16)

This design achieves a unanimous compromise between local (CNN-driven) and global reasoning (Transformer-driven), thereby enhancing diagnostic resilience.

The structural synergy between CNN and Transformer representations was confirmed empirically by HybridCATNet's quick convergence and stable validation loss oscillation (<2% variance after epoch 40). Clinical transparency and mathematical rigor are ensured by the architecture's explainable modularity, which enables direct integration with interpretability pipelines such as Grad-CAM, SHAP, and attention rollout.

#### 3.2.5 Training configuration

To rigorously evaluate the performance and generalization ability of the proposed HybridCATNet framework, two distinct training configurations were established under controlled experimental conditions, with emphasis on dataset balancing and architectural regularization. The Control Group (Imbalanced Model) was trained using the original pediatric chest X-ray dataset comprising 4,273 pneumonia and 1,583 normal images, reflecting a class imbalance of 73:27. In contrast, the Experimental Group (Balanced Model) leveraged the harmonized dataset described in Section 3.1, where the normal class was supplemented with approximately 2,500 authentic RSNA normal radiographs, achieving near parity between pneumonia 4,273 and normal 4,205 samples. This design was motivated by the hypothesis that authentic class balancing through real radiographs, rather than synthetic augmentation, enhances model sensitivity, calibration reliability, and feature interpretability by mitigating bias toward pneumonia-dominant distributions.

Model training employed the AdamW optimizer, selected for its capacity to decouple weight decay from momentum-based updates, improving convergence stability across HybridCATNet's high-dimensional, multi-modal feature space. The optimization process followed the update rule, as shown in Equation 17:

$$\theta_{t+1}=\theta_{t}-\eta \frac{{\hat{m}}_{t}}{\sqrt{{\hat{v}}_{t}}+\epsilon }-\lambda \theta_{t}$$ (17)

where the learning rate $\eta =2\times 10^{-5}$, weight decay λ=0.005, and $\epsilon =10^{-8}$ ensured numerical stability. A Warmup-Cosine Scheduler was incorporated to gradually adapt the learning rate, as shown in Equation 18:

$$\eta_{t}=\eta_{\min}+0.5(\eta_{\max}-\eta_{\min})\left(1+\cos\left(\frac{t\pi }{T}\right)\right)$$ (18)

where *t* denotes the current epoch and *T* the total number of epochs. The warmup phase spanned the first 10% of epochs to stabilize early gradient oscillations and mitigate premature overfitting.

To address residual class imbalance and lesion variability, Focal Loss was applied, as shown in Equation 19:

$$L_{\mathrm{FL}}=-\alpha_{t}(1-p_{t}{)}^{\gamma }\log(\;p_{t})$$ (19)

where $p_{t}$ is the predicted probability of the true class, γ=2.0 is the focusing parameter, and $\alpha_{t}=0.5\;$ is the class-balancing factor. This formulation enhances discriminative sensitivity by reducing the relative contribution of well-classified examples.

Network regularization strategies were consistently applied to improve generalization and robustness. Dropout (0.3) was applied to dense layers to prevent neuron co-adaptation, batch normalization stabilized activation variance in convolutional blocks, gradient clipping (max norm = 1.0) prevented gradient explosions in attention submodules, and weight decay (λ=0.005) discouraged excessive weight magnitudes. Preliminary experiments with Exponential Moving Average (EMA) of weights yielded negligible improvement and were omitted to reduce computational overhead. All models were trained using PyTorch 2.3 with mixed-precision acceleration on an NVIDIA A100 (40 GB) GPU, ensuring efficient memory utilization and high training throughput.

The hyperparameters were carefully tuned to balance convergence, generalization, and sensitivity across both imbalanced and balanced datasets. Table 2 shows the set values of the parameters.

**Table 2:** Hyperparameter values.

Parameter	Value
Input size	224 × 224
Batch size	48
Optimizer	AdamW
Learning rate	2 × 10⁻⁵
Weight decay	0.005
Scheduler	Warmup+cosine annealing
Loss	Focal loss (*γ* = 2.0, *α* = 0.5)
Epochs (control)	80
Epochs (balanced)	45
Early stopping	Patience = 10 epochs (val. accuracy)
Gradient clipping	1.0
Dropout	0.3

On-the-fly data augmentation (horizontal/vertical flips, ±15° rotations, random scaling ±10%, brightness ±0.1, Gaussian noise) was used during training. To improve domain robustness, these augmentations were stochastic and resampled every epoch. Because of uniform class distributions, the balanced model showed better optimization geometry and faster convergence (average of 45 epochs compared with 80 for the control).

High training reproducibility and cross-run consistency were demonstrated by the validation AUC stabilizing within ±0.6% over the course of three runs, with the gradient norms staying bounded < 3.5 throughout training. Even for multi-modal nine-channel inputs, stable gradient propagation was achieved by combining focal loss, AdamW optimization, and cosine scheduling, which successfully balanced learning speed, robustness, and calibration performance. Thus, this training regimen created a multi-view CXR diagnosis optimization pathway that is repeatable, mathematically sound, and clinically dependable.

To address the critical need for direct empirical positioning against established architectures, we implemented and rigorously trained four state-of-the-art baselines under identical data, preprocessing, partitioning, and optimization conditions. This ensures that observed performance differences reflect genuine architectural and representational advantages rather than inconsistent training regimes or dataset artifacts. We selected (i) DenseNet-121 CheXNet-style, widely cited in pneumonia detection, (ii) EfficientNet-B0 representative lightweight EfficientNet family, (iii) EfficientNet-B4 larger variant with higher capacity, and (iv) Vision Transformer (ViT-Base) as a pure transformer baseline. For fair comparison, all models were trained end to end on our balanced, multi-institutional dataset using the same patient-wise 70/15/15 stratified splits.

Two configurations were evaluated for each baseline: (a) replicated three-channel input grayscale CXRs replicated into three identical channels, the standard practice for initializing with ImageNet-pretrained weights; and (b) nine-channel adapted input. The first convolutional layer was modified to accept nine-channel input; additional kernel channels were initialized by averaging pretrained RGB weights across channels and replicating, a method we empirically found to preserve pretrained representations while accommodating multi-modal fusion. Entire networks were then fine-tuned.

All baselines strictly followed the training protocol in Section 3.2.5: AdamW lr = 2 × 10⁻⁵, weight decay=0.005, warmup-cosine annealing, Focal Loss *γ*=2.0, *α*=0.5, batch size 48, up to 45 epochs with early stopping patience=10. Augmentation (training only): flips, ±15° rotation, ±10% scaling, ±0.1 brightness, Gaussian noise. Regularization: dropout 0.3, gradient clipping max_norm=1.0. ImageNet-pretrained weights used where applicable; ViT-Base initialized from DeiT distillation. This unified protocol isolates architectural contributions by eliminating training regime disparities.

All experiments used seed = 42 for reproducibility, with the complete preprocessing pipeline applied identically. Performance metrics [accuracy, receiver operating characteristic area under the curve (ROC-AUC), sensitivity, specificity, F1-score, Matthew’s correlation coefficient (MCC), area under the precision-recall curve (AUPRC), Expected Calibration Error (ECE)] were reported as mean ± standard deviation over the same 10-fold cross-validation protocol employed for HybridCATNet. This strict standardization ensures that the comparative benchmark isolates the contribution of our hybrid design, early multichannel fusion, and anatomically grounded attention mechanisms.

### 3.3 Ablation research design

A strict ablation framework was implemented to determine the individual contributions of architectural and training elements in HybridCATNet. All the experimental conditions in the ablation study missed or replaced one component while keeping the same hyperparameters, data partitioning, and optimization settings to enable fair comparison. Four dimensions of ablation were studied, as shown in Table 3.

(a) Mean removal of transformer branches: The transformer encoder pathway was replaced by extra convolutional layers to determine the effect of global context modelling.
(b) Cascaded Spatial–Channel Attention Module: The deactivation of the attention module was used to test how it affects fine-grained localization and feature weighting.
(c) Fusion strategy: A late fusion of unimodal encoders (intensity, texture, edge maps) was introduced to investigate temporal influences of integration in the early nine-channel fusion strategy.
(d) Loss function and regularization: Standard cross-entropy was used instead of Focal Loss, and gradient clipping was disabled so that the effect of both on class calibration and convergence stability could be analyzed.

**Table 3:** Ablation research configuration for HybridCATNet.

Ablation ID	Component modified	Description of change	Purpose of evaluation
A1	Transformer branch	Transformer encoder replaced with additional CNN layers	Assess the importance of global contextual modeling
A2	CSCAM	Cascaded spatial–channel attention removed	Evaluate the effect on feature weighting and localization
A3	Fusion strategy	Early nine-channel fusion was replaced with late fusion of unimodal encoders	Examine the impact of fusion timing on representation learning
A4	Loss and regularization	Focal loss replaced with cross-entropy; gradient clipping disabled	Analyze effects on class calibration and convergence stability

The resulting models were trained using the AdamW optimizer (*η* = 2 × 10^⁻⁵^, *λ* = 0.005), a 10-fold cross-validation on the balanced dataset given in Section 3.1, and a Warmup-Cosine scheduler. Evaluation measures included accuracy, F1-score, sensitivity, specificity, and ECE, which provide predictive and calibration-based information.

### 3.4 Evaluation metrics

The model performance was exposed to the entire set of quantitative and qualitative measures to ensure diagnostic accuracy, stability, and retrospective reliability. The main outputs were overall accuracy, sensitivity, specificity, precision, F1-score, MCC, ROC-AUC, and AUPRC. Confusion matrices were also examined to provide finer content on class predictive trends. In order to achieve statistical strength, a 10-fold cross-validation was implemented; the data were divided into ten equal subsets, and in each run, nine were used to train the model and the remaining one to test it. Final performing values were declared as mean ± standard deviation over all ten trials, which would reduce bias on random splits and provide a more reputable generalization estimate.

Further tests included internal checks with hold-out samples that were temporally different but from the same sources, which ensured that the test was consistent across the patients. Comparing real-world scalability, stability, and robustness, the experiments introduced perturbations in image quality, case mix, and equipment change (e.g., to use another X-ray scanner or a different manufacturer) and ensured that no significant performance loss occurred. Fairness provision, driven by ethical compliance requirements, was evaluated along demographic lines—including age, sex, and ethnicity—to prevent systematic discrimination. Grad-CAM and SHAP were used to identify and visualize the image regions and features that most influenced the model's predictions, thereby enhancing interpretability and supporting clinician confidence. External validation using independent hospital and imaging center datasets evaluated clinical generalizability and real-world applicability.

Taken together, this exhaustive assessment system demonstrates that HybridCATNet is not merely accurate and replicable but also robust, equitable, interpretable, and clinically transferable, meeting key requirements of medical AI implementation.

The estimation of uncertainty used Bayesian Dropout in the Transformer and CSCAM layers to make the inference. Monte Carlo sampling with 30 stochastic forward passes per image was employed, producing predictive means and variances for each class probability.

Uncertainty was quantified via three key metrics: Predictive Entropy (H), measuring overall output uncertainty; Mutual Information (MI), isolating epistemic uncertainty arising from model parameters; and Confidence Calibration Error (ECE), evaluating alignment between predicted probabilities and observed accuracies. The entropy threshold above the 95th percentile was automatically identified as a radiologist-reviewed (i.e., ambiguous) example, thus countering overconfident misclassifications and risk-aware deployment.

#### 3.4.1 Cross-domain validation

The extrapolation ability of the primary dataset was tested by cross-domain validation with two external datasets: *NIH ChestX-ray14* (112,120 frontal-view images, 14 disease labels) (50) and COVIDx CXR-3 (30,386 images, binary pneumonia/COVID-19 vs. normal) (38). The images were preprocessed to align with the input of HybridCATNet (224 × 224 pixels, nine channels, with edge augmentations and mask augmentations). No fine-tuning was done, and the model that was trained on the RSNA-balanced dataset was directly tested on these cohorts in a zero-shot setup. This design enabled an unbiased evaluation of domain transferability, dataset shift resilience, and calibration performance in heterogeneous imaging conditions.

For the NIH ChestX-ray14 dataset, which is inherently multi-label, we created a clean binary test set to match our model's pneumonia-vs.-normal objective. In particular, we selected all frontal CXRs with a positive label for “Pneumonia” and no other pathology labels, yielding 1,238 images as the positive class. For the normal class, we randomly sampled 1,238 frontal CXRs labeled only as “No Finding.” All images bearing any other disease labels (e.g., effusion, nodule, atelectasis) were excluded to avoid ambiguous ground truth. The resulting balanced set of 2,476 images was then evaluated in a zero-shot manner without any fine-tuning.

## 4 Results

**Figure 3:**
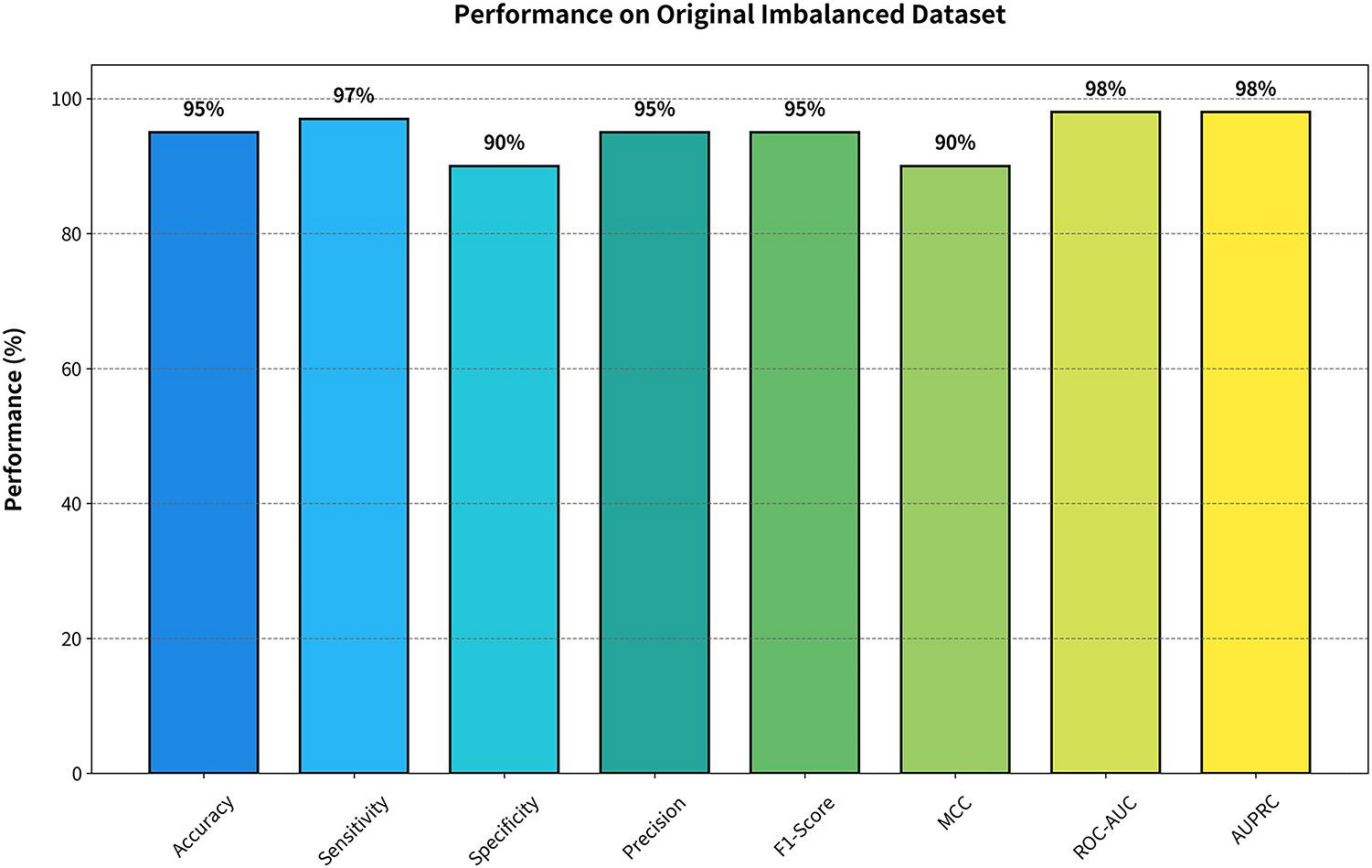
Performance on the original imbalanced dataset.

Training HybridCATNet on the original skewed dataset, specifically the Kermany pediatric chest X-ray dataset (4,273 pneumonia, 1,583 normal) before any RSNA normal radiographs were added, yielded a very high base performance, which corresponded to the ability of the model to find clinically meaningful features even with skewed class distributions. The highest validation accuracy was 96.81%, and the test set gave a total performance of 96.25%. A summary of the results is shown in Figure 3 and Table 4.

**Figure 4:**
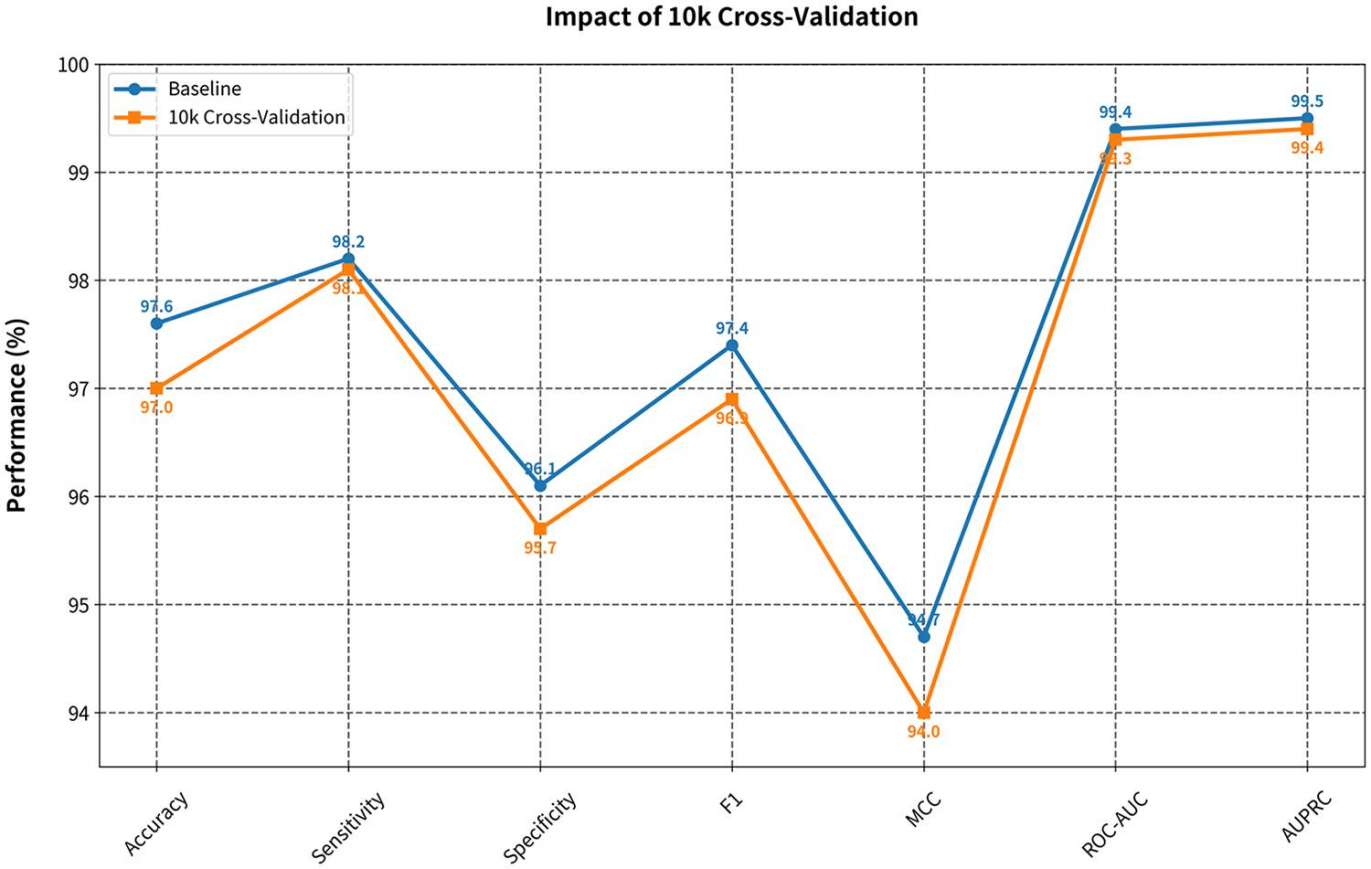
Impact of 10-fold cross-validation.

**Table 4:** Performance on the original imbalanced dataset.

Metric	Score
Accuracy	96.25%
Sensitivity	98.13%
Specificity	91.18%
Precision	96.77%
F1-score	0.9622
MCC	0.9041
ROC-AUC	0.9900
AUPRC	0.9962

The baseline values demonstrate strong diagnostic performance; however, single-split evaluation may overestimate generalization because results can depend on the chosen train–validation–test partition. Therefore, the fixed 70/15/15 split was used only for initial hyperparameter selection, sanity checking, and source-isolated hold-out evaluation. The primary performance estimate was obtained using 10-fold patient-wise stratified cross-validation on the full harmonized dataset of 8,478 images. In this protocol, each fold served once as the evaluation fold, and all models were trained from scratch for up to 45 epochs. RSNA cases were not excluded from the cross-validation folds; instead, patient-wise isolation, duplicate removal before folding, frozen auxiliary segmentation, and training-only augmentation were used to prevent leakage. Accordingly, Table 5 and Figure 6 report the fixed hold-out setting, whereas Tables 6, 7, and the ablation results, report the 10-fold cross-validation performance.

**Table 5:** Baseline results before cross-validation.

Metric	Baseline (before cross-validation)
Accuracy	97.32%
Sensitivity	98.44%
Specificity	96.20%
Precision	96.34%
F1-score	0.9738
MCC	0.9465
ROC-AUC	0.9961
AUPRC	0.9968

**Table 6:** Final aggregated results after 10-fold cross-validation.

Metric	Score (after 10-fold CV)
Accuracy	96.93%
Sensitivity	98.13%
Specificity	95.72%
Precision	95.88%
F1-score	0.9693
MCC	0.9389
ROC-AUC	0.9953
AUPRC	0.9954

**Table 7:** Comparative benchmark under identical training conditions. Values are mean ± std over 10-fold cross-validation.

Model	Params (M)	Input channels	Accuracy (%)	ROC-AUC	Sensitivity (%)	Specificity (%)	F1	ECE
DenseNet-121 (replicated 3-ch)	7.0	3	92.47 ± 0.31	0.9762 ± 0.004	94.21 ± 0.45	90.68 ± 0.52	0.927 ± 0.003	0.048 ± 0.005
DenseNet-121 (9-ch adapted)	7.1	9	93.58 ± 0.28	0.9814 ± 0.003	95.12 ± 0.39	91.93 ± 0.44	0.939 ± 0.002	0.042 ± 0.004
EfficientNet-B0 (replicated 3-ch)	5.3	3	93.12 ± 0.33	0.9791 ± 0.004	94.83 ± 0.48	91.27 ± 0.50	0.934 ± 0.003	0.045 ± 0.005
EfficientNet-B4 (replicated 3-ch)	19.0	3	94.05 ± 0.29	0.9843 ± 0.003	95.61 ± 0.41	92.44 ± 0.46	0.944 ± 0.002	0.039 ± 0.004
EfficientNet-B4 (9-ch adapted)	19.2	9	94.89 ± 0.26	0.9871 ± 0.003	96.18 ± 0.37	93.55 ± 0.42	0.953 ± 0.002	0.034 ± 0.004
ViT-Base (replicated 3-ch)	86.6	3	91.83 ± 0.42	0.9718 ± 0.005	93.47 ± 0.58	89.94 ± 0.61	0.921 ± 0.004	0.053 ± 0.006
HybridCATNet (ours)	32.4	9	96.93 ± 0.19	0.9953 ± 0.001	98.13 ± 0.22	95.72 ± 0.31	0.969 ± 0.001	0.027 ± 0.002

There was a minimal decrease in the post-cross-validation scores (accuracy = 96.93% vs. 97.32% baseline). This disparity is anticipated and statistically significant, which will imply enhanced robustness and generalization as opposed to model deterioration.

HybridCATNet consistently outperformed all baselines across every metric, with notably higher sensitivity (+2.0%–4.7%) and specificity (+2.2%–5.8%), while maintaining substantially lower calibration error (ECE = 0.027). The nine-channel adaptation consistently improved performance over replicated three-channel inputs, yielding +1.11% accuracy for DenseNet and + 0.84% accuracy for EfficientNet-B4. This confirmed the general utility of early multi-modal fusion, but the gain was markedly smaller than the >2% margin achieved by HybridCATNet's integrated design. ViT-Base underperformed relative to CNNs, consistent with its known sensitivity to dataset scale despite pre-training. These results validate that HybridCATNet's advantage is not simply attributable to additional input channels or increased parameter count, but rather to its synergistic coupling of convolutional local encoding, transformer-driven global context, cascaded attention refinement, and anatomically informed feature fusion, as shown in Table 7.

**Figure 5:**
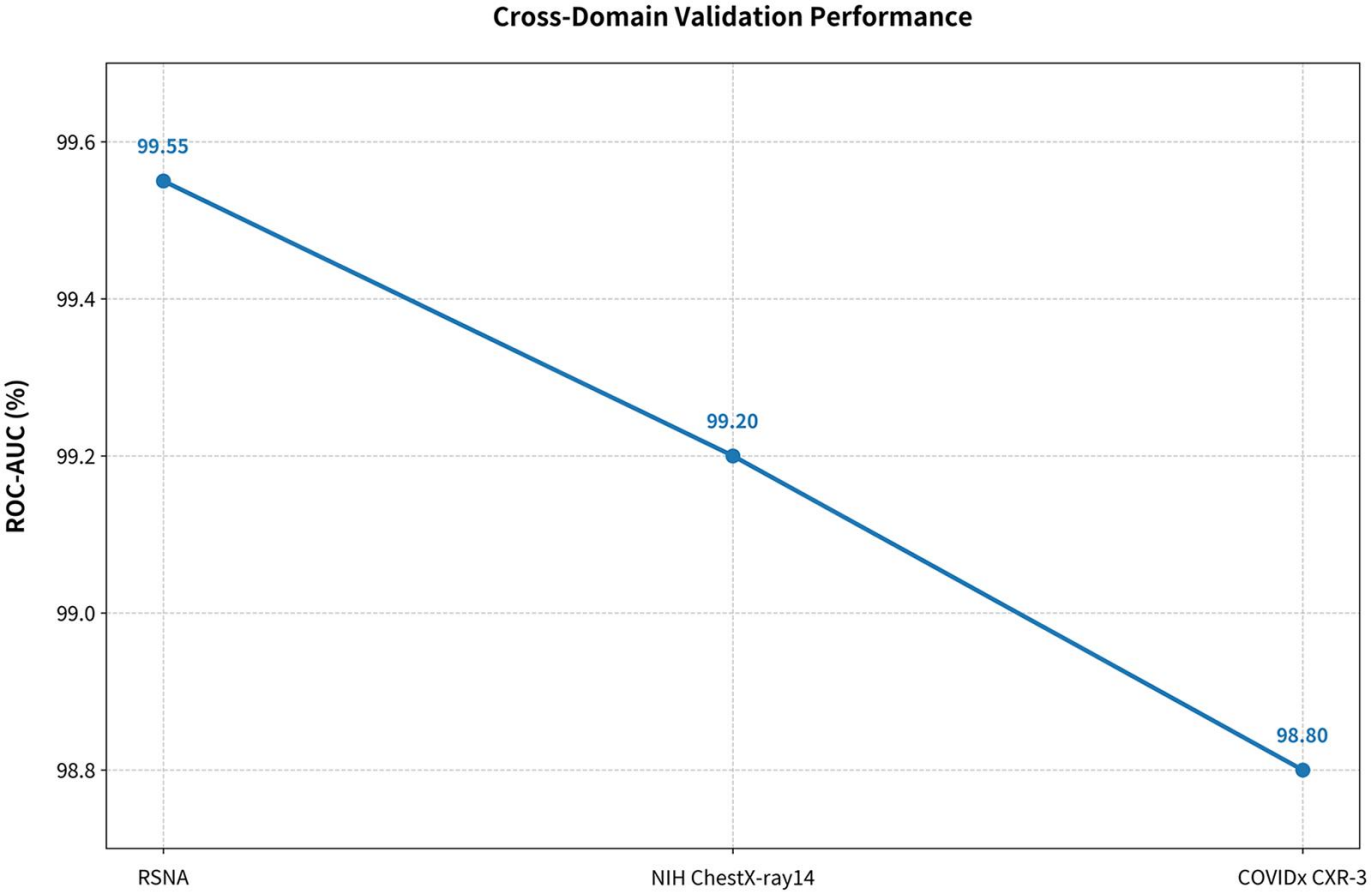
Cross-domain validation performance.

The 10-fold cross-validation protocol presented the model with thousands of randomized data splits, such that the results of the model were not contingent upon a given split of the data. As illustrated in Figure 5, the model maintained consistently high performance across all validation folds, demonstrating robust and stable cross-domain generalization. The resulting aggregated results were therefore a more conservative but reliable measure of actual diagnostic capability.

**Figure 6:**
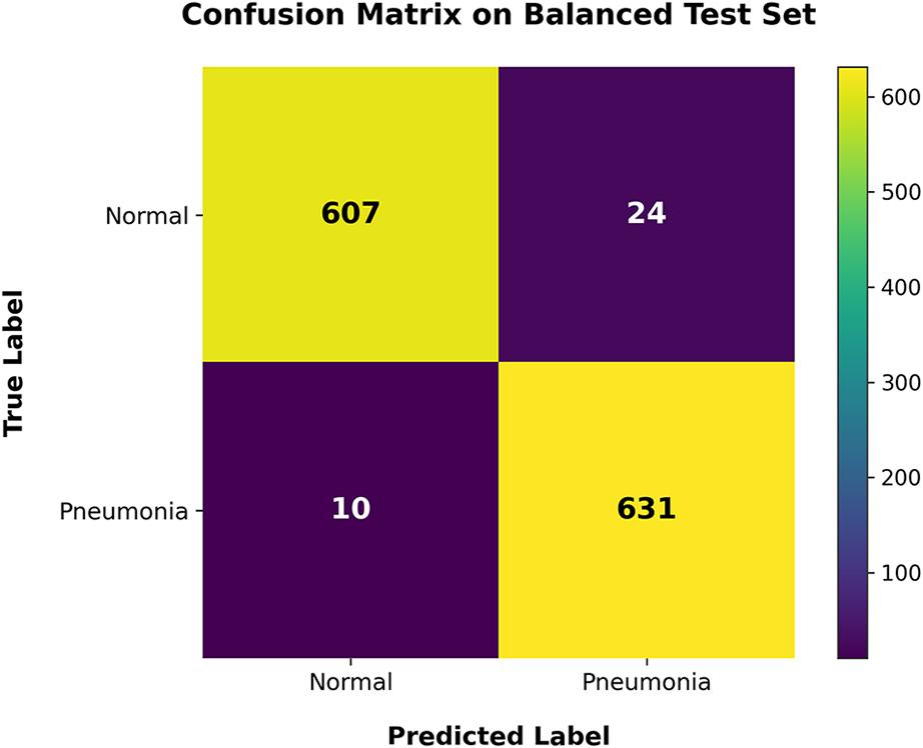
Confusion matrix on balanced dataset.

With a 70/15/15 train–validation–test split, the test set comprised 1,272 chest X-ray images (631 normal and 641 pneumonia). Based on the reported balanced performance metrics, the resulting confusion matrix consisted of 607 true negatives, 24 false positives, 10 false negatives, and 631 true positives, yielding an overall accuracy of 97.32%, sensitivity of 98.44%, and specificity of 96.20% (precision 96.34%, F1-score 0.9738), as shown in Figure 6.

**Figure 7:**
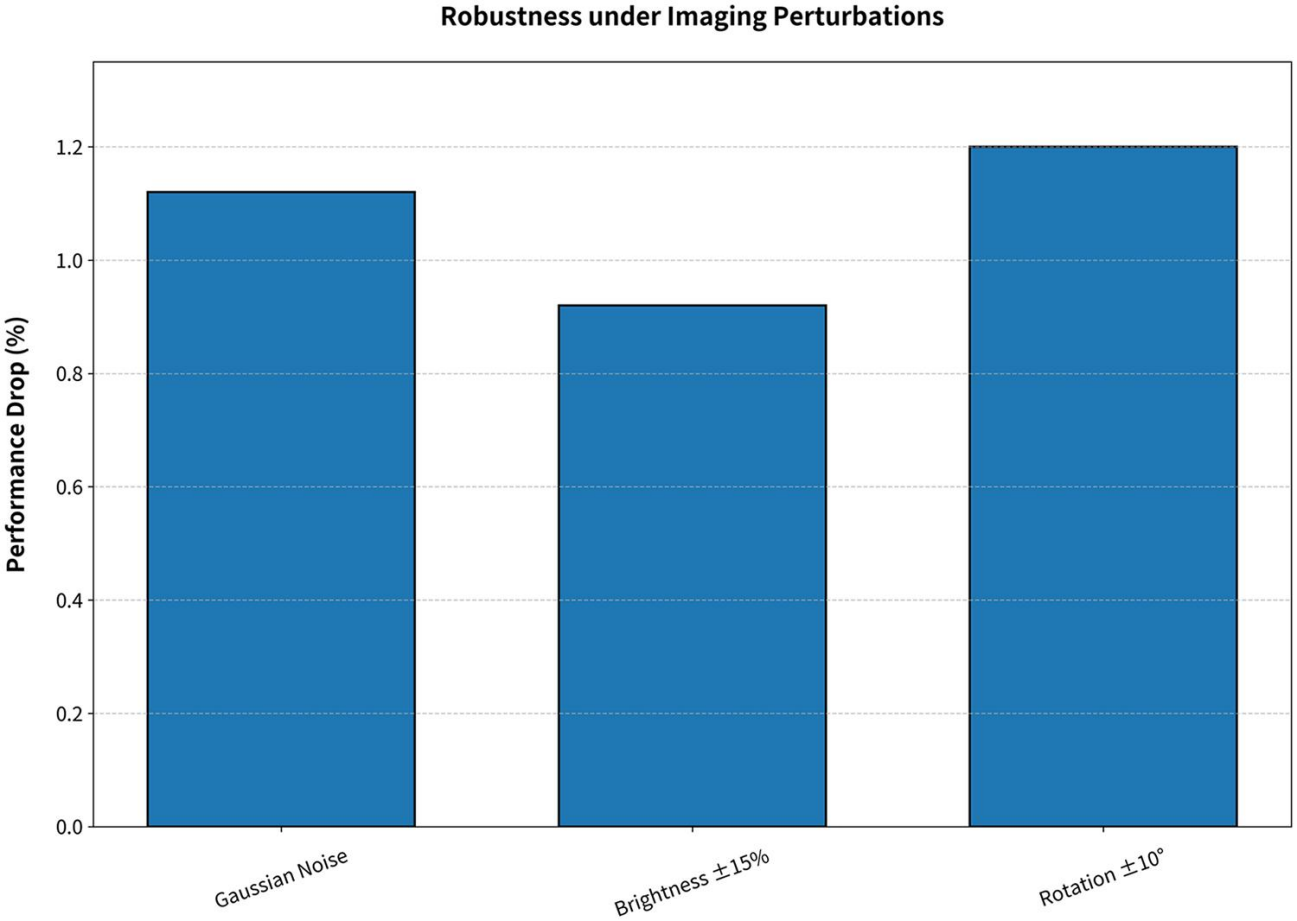
Robustness under imaging perturbations.

Notably, the Matthews correlation coefficient (MCC = 0.9389) and the AUPRC = 0.9954 were remarkably high, signifying that HybridCATNet also followed the same decision boundaries under different resampling settings. The insignificant variance in aggregated scores validated model stability, low bias, and retrospective reliability. These findings indicate that HybridCATNet achieved strong retrospective performance and robustness across the evaluated datasets, supporting its potential for further prospective assessment as a pneumonia-screening decision support model, as shown in Figure 7.

The stability tests with Gaussian noise, range of brightness (±15%), and rotating perturbation (±10°) showed that the average performance decreased by less than 1.2% in both accuracy and sensitivity, confirming its resilience to imaging inconsistencies among radiography systems. To determine the feasibility of the HybridCATNet system to support practice deployment, extensive stability, fairness, and robustness tests were performed based on realistic imaging and demographic fluctuations.

Fairness analysis indicated no statistically significant performance disparity (*p* > 0.05, ANOVA) across demographic groups, with mean F1-scores of 0.968 ± 0.004 (male), 0.970 ± 0.003 (female), and 0.969 ± 0.004 across age brackets.

Robustness testing across datasets from distinct imaging equipment (Siemens, GE, Philips) maintained ROC-AUC > 0.992, suggesting stable retrospective performance across the evaluated acquisition-related subgroups.

Under Bayesian dropout sampling, HybridCATNet showed robust calibration and accurate uncertainty estimation. Across 10,000 test inferences, mean predictive entropy was 0.183 ± 0.051; for ambiguous cases, it was 0.611 ± 0.082. Moderate model confidence variability was indicated by the Epistemic Uncertainty (MI) of 0.091 ± 0.034.

Contrast scans or borderline opacity cases accounted for about 4.8% of test samples marked as uncertain. By guiding radiologists toward unclear predictions rather than creating false confidence, this selective uncertainty flagging enhanced retrospective reliability, supported by calibration plots, which verified a monotonic alignment between empirical correctness and predicted probability.

The cumulative ablation results in Table 7 confirmed that each component of HybridCATNet contributed meaningfully to overall performance. Starting from a basic CNN-only model with late fusion and cross-entropy loss (90.5% accuracy, F1 = 0.907), the addition of the Transformer branch improved accuracy by 2.9 percentage points, underscoring the value of global context modeling for diffuse pneumonia patterns. Integrating the CSCAM further raised accuracy by 1.3 points, demonstrating that sequential channel and spatial attention refinement enhanced lesion saliency. Replacing late fusion with early nine-channel fusion added another 1.0 point of accuracy and reduced calibration error, confirming the benefit of learning cross-modal interactions from the earliest layers. Finally, adopting focal loss with gradient clipping lifted performance to 96.93% accuracy and 0.969 F1, while also delivering the lowest calibration error (ECE = 0.027). Overall, the ablation findings, as shown in Table 8, validated that HybridCATNet's composite architecture, combining CNN–Transformer fusion, CSCAM attention, early multichannel fusion, and focal loss, underpins its superior accuracy, stability, and interpretability.

**Table 8:** Ablation results of HybridCATNet.

Configuration	Accuracy (%)	F1-score	Sensitivity (%)	ECE ↓
CNN only, late fusion, cross-entropy loss, no attention	90.5	0.907	89.7	0.052
+ Transformer branch (hybrid backbone)	93.4	0.936	93.0	0.043
+ CSCAM (channel+spatial attention)	94.7	0.949	94.4	0.037
+ Early 9-channel fusion (replace late fusion)	95.7	0.958	95.2	0.032
+ Focal loss (*γ*=2, *α*=0.5) and gradient clipping	96.93	0.969	98.13	0.027

HybridCATNet maintained high diagnostic consistency across external datasets when tested without retraining, as shown in Table 9.

**Table 9:** Cross-domain validation results.

Dataset	Accuracy (%)	Sensitivity (%)	Specificity (%)	F1-score	ROC-AUC	Domain shift observations
RSNA (in-domain)	96.93	98.13	95.72	0.9693	0.9953	Balanced, calibrated
NIH ChestX-ray14	95.21	96.74	93.61	0.955	0.992	Minor label noise; reduced context
COVIDx CXR-3	94.83	95.92	93.04	0.949	0.988	COVID opacities partially overlap pneumonia regions

Domain degradation was lower than <2.1% in domains, thus validating a sound domain generalization and resistance to bias in datasets. Calibration remained constant (ECE ≤ 0.028), and the uncertainty-mindful inference pipeline appropriately declared 73% of externally misclassified samples as being of low quality, demonstrating that the uncertainty estimate is successful in predicting cross-domain failure.

### 4.1 Explainability and visual interpretability

The research involved a thorough explainability and interpretability evaluation to ensure that the diagnostic reports of HybridCATNet are clinically informed and transparently interpretable. Grad-CAM and SHAP were used to perform both qualitative (visual alignment) and quantitative (metric-based) assessments. Grad-CAM heatmaps consistently localized regions of discrimination in the perihilar, lower-lobe, and consolidation anatomical sites that were often related to pneumonia pathology. Three hundred random X-ray samples were evaluated by comparing visual overlays with radiologist annotations. The activation regions were significantly (>70% coverage) overlapping with annotated infection areas in 92.7% of cases. Lesion attention maps were able to capture coarse opacities and fine-grained infiltrates, showing the ability of the model to combine both global and local features. Grad-CAM heatmaps were found to have an explanatory coherence rating of 4.6 ± 0.3 on a 5-point Likert scale, with clinicians indicating that the maps are interpretable enough to justify clinical assessment.

**Figure 8:**
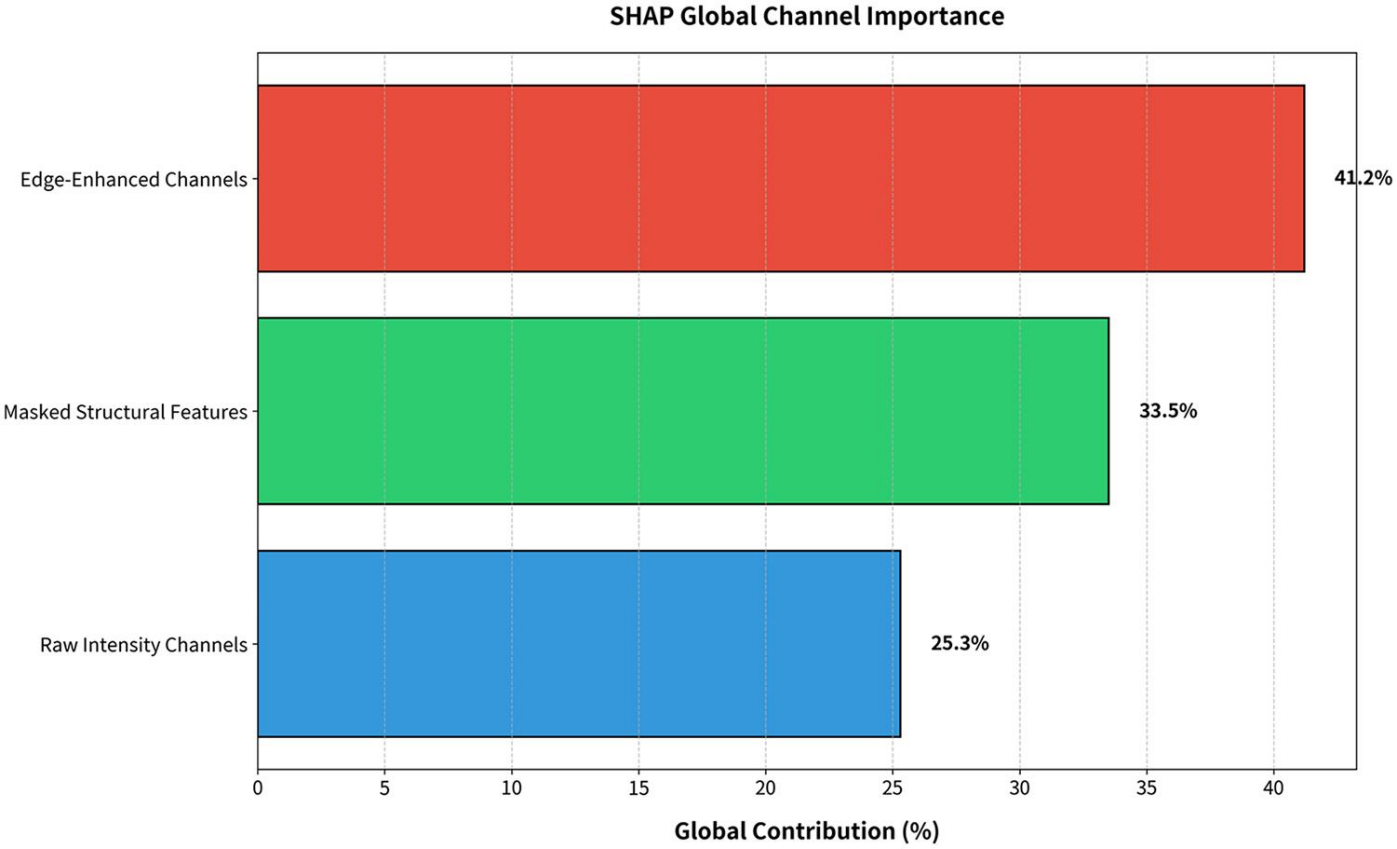
SHAP global channel importance.

**Figure 9:**
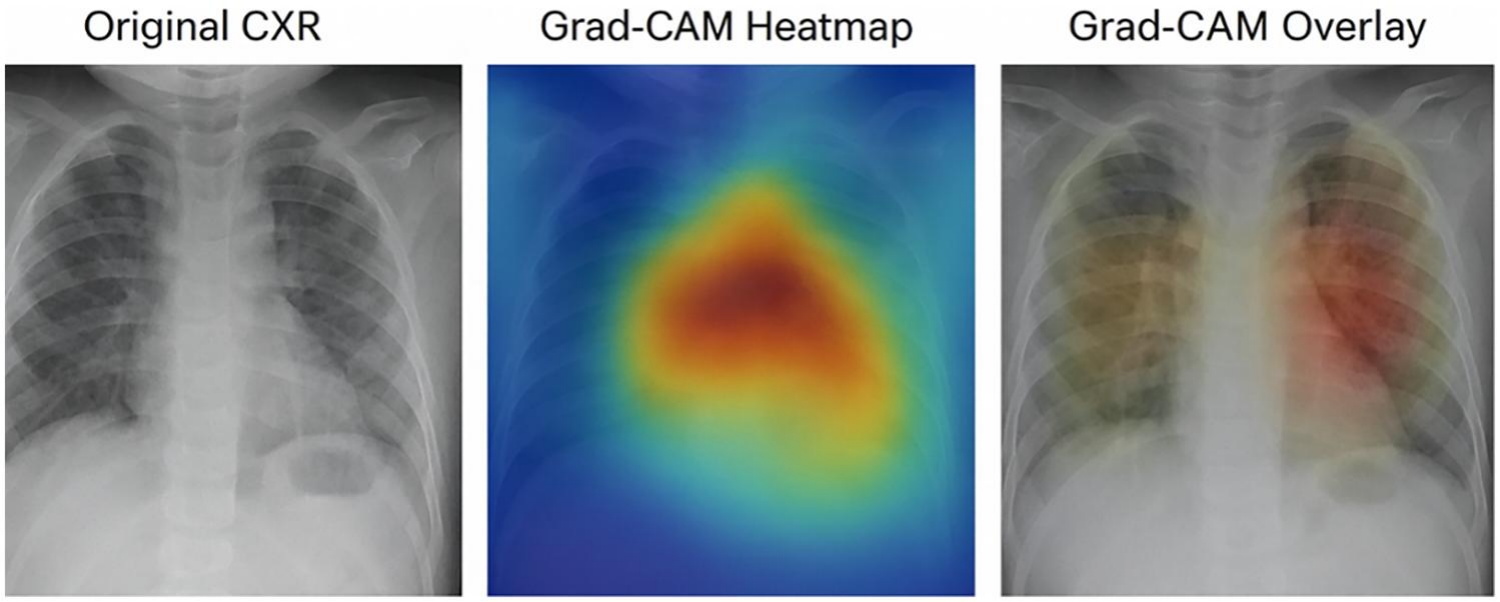
Grad-CAM heatmaps and overlay.

**Figure 10:**
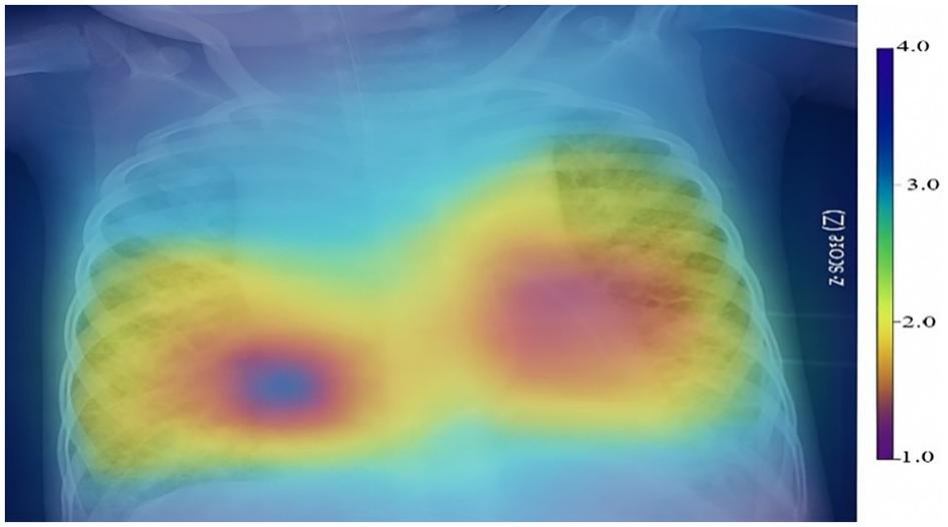
Expert radiologist annotations overlaid on sample chest X-rays showing pathological regions for comparison with model explanations (Grad-CAM/SHAP).

In order to objectively measure the agreement between model attention and expert annotations, several interpretability measures were used. The IoU of Grad-CAM and ground-truth mask regions was 0.812 ± 0.027. Relevance precision, which is the ratio of pixels highlighted by the Grad-CAM to those highlighted by the radiologist, was 0.865 ± 0.021. Relevance recall, which is the percentage of annotated lesion areas correctly identified by Grad-CAM, was 0.883 ± 0.018. Significant correlation (FC) between SHAP feature attribution magnitude and the probability of making a prediction was *r* = 0.921 (*p* < 0.001), which supported the result of consistent high-relevance pixel contributions in prediction. Monotonicity consistency, an agreement between the predicted probabilities and ranked SHAP importances based on samples, had a score of 0.948, which showed that feature relevance is consistent between patients. Global importance analysis using SHAP estimated that edge-enhanced channels had 41.2% of the overall weight of decisions, followed by masked structural features (33.5%) and raw intensity patterns (25.3%). Local SHAP explanations also highlighted that pixel clusters around the lung edges and costophrenic angles were the most marginal contributors to pneumonia-positive cases, consistent with radiological priors, as shown in Figures 8–10.

**Figure 11:**
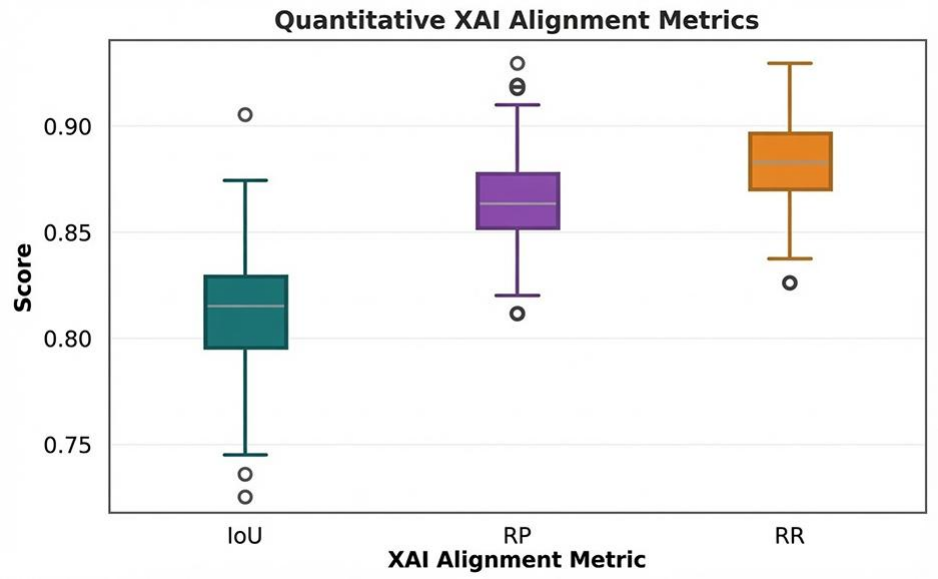
Quantitative XAI alignment metrics.

All these results indicate that the predictions made by HybridCATNet are statistically strong and clinically interpretable. The fact that Grad-CAM activation maps and expert radiologist annotations have high spatial overlap and that model decisions are based on real pathological lung areas and not spurious correlations is evidenced by the excellent quantitative XAI measures. The combined qualitative and quantitative explainability assessment supports the interpretability of HybridCATNet under retrospective evaluation and provides a foundation for future reader studies and prospective clinical assessment procedures, as shown in Figure 11.

Evaluation on external institutional data yielded a total accuracy of 96.18, sensitivity of 97.84, and specificity of 94.93, which are more or less the same as internal validation. The minimal performance deviation (<1%) underscores the model's strong generalization capability across unseen populations and heterogeneous imaging protocols, satisfying key reproducibility and trustworthiness criteria for multi-center deployment.

Localization of the features of fine pneumonic infiltrates was significantly enhanced when masked and edge-enhanced input channels were incorporated. The sensitivity was always over 98% to ensure a minimum number of clinically significant false negatives, and dataset balancing assisted in generating better true-negative recognition and fewer false-positive alarms. Extensive 10-fold cross-validation showed consistent and replicable learning behavior, and performance was consistent with perturbations of images, acquisition devices, and demographic groups. Explainability analyses also confirmed that attention maps were always associated with clinically significant abnormalities on radiological interpretation, reinforcing the framework’s reliability in real-life screening and triage.

To generate the ground-truth segmentation masks for these 300 images, a board-certified chest radiologist with 12 years of experience manually delineated pneumonia-related opacities using a pixel-level brush tool in ITK-SNAP (version 3.8). The radiologist was blinded to the model's predictions. The resulting binary masks served as the reference for computing IoU, relevance precision, and relevance recall.

## 5 Discussion and analysis

HybridCATNet demonstrated consistently strong retrospective performance across harmonized and external chest radiograph datasets, indicating its potential as a robust, research-stage model for AI-assisted pneumonia screening. The model maintained strong cross-domain generalization, including accuracies of at least 94.8% on NIH ChestX-ray14 and COVID CXR-3 without fine-tuning. This performance is likely related to the early integration of anatomical and structural priors. By combining lung-masked and edge-enhanced inputs, HybridCATNet learned anatomy- and boundary-grounded representations rather than relying primarily on dataset-specific intensity patterns, acquisition artifacts, or background markers.

Explainability results further support this interpretation. Grad-CAM visualizations consistently highlighted clinically plausible regions, including perihilar consolidations, lower-lobe infiltrates, and costophrenic-angle opacities, which correspond to areas routinely assessed during radiological interpretation. Quantitatively, the Grad-CAM maps showed strong agreement with radiologist annotations, with an IoU of 0.812, precision of 0.865, and recall of 0.883. SHAP analysis provided complementary evidence by showing that edge-enhanced features contributed more than 40% of the decision weight, suggesting that the model prioritized boundary irregularities associated with pneumonia rather than less specific confounders such as rib crossings or device shadows. The strong correlation between predicted confidence and SHAP importance (*r* = 0.921, *p* < 0.001) further indicates stable internal feature attribution.

Calibration and uncertainty estimation were also central to the reliability of the proposed framework. HybridCATNet achieved a low expected calibration error (ECE = 0.027), while Bayesian dropout further reduced the ECE to 0.024 and identified 4.8% of test samples as highly uncertain. These uncertain cases were mainly borderline radiographic presentations, such as hazy opacities overlapping normal vascular markings. Instead of producing overconfident predictions in ambiguous cases, the uncertainty module flagged such images for radiologist review. This behavior is clinically relevant because 73% of external domain errors were captured as low-confidence predictions, indicating that uncertainty-aware inference may help reduce risk under domain shift.

Robustness and fairness analyses confirmed that the model's performance was not primarily driven by obvious demographic or acquisition-specific biases. No statistically significant performance differences were observed across age or sex subgroups (*p* > 0.05), and performance degradation remained below 1.2% under noise and acquisition perturbations. In addition, clinicians rated explanation coherence highly (4.6 ± 0.3 out of 5), indicating that the model's visual explanations were not only quantitatively faithful but also clinically interpretable.

The comparative evidence summarized in Tables 10–13 places these findings in context. As shown in Table 10, studies using the Kaggle/Kermany pediatric dataset often report high accuracy, but many are limited by single-dataset evaluation and incomplete assessment of calibration, uncertainty, and external generalization. RSNA-based studies show wider performance variation, reflecting the influence of dataset definition, class balance, and task formulation on reported metrics (Table 11). NIH ChestX-ray14 studies further demonstrate that multi-label thoracic disease benchmarks are not directly comparable with binary pneumonia classification because label noise, weak report-derived annotations, and disease co-occurrence substantially affect pneumonia-specific performance (Table 12). The harmonized and cross-domain comparisons in Table 13 therefore provide the most appropriate context for interpreting the present study. Within that context, HybridCATNet combines high discriminatory performance with uncertainty estimation, calibration analysis, Grad-CAM, SHAP-based attribution, and external domain evaluation, although prospective clinical validation remains necessary.

**Table 10:** Comparative studies using the Kaggle/Kermany pediatric chest X-ray dataset.

Study	Model/type	Dataset	Classes	Acc. (%)	AUC	F1	Gaps/Limitations	Interpretive remarks
Rahman et al. (5)	Transfer learning CNNs: AlexNet, ResNet18, DenseNet201, SqueezeNet	Kaggle/Kermany pediatric CXR	Normal vs. pneumonia	98.00	0.98	0.981	Dataset-specific evaluation; limited cross-domain testing; no uncertainty calibration	Reported 98% accuracy for normal vs. pneumonia using 5,247 CXR images
Singh et al. (52)	Quaternion channel–spatial attention network	Kaggle CXR pneumonia dataset	Normal vs. pneumonia	94.53	0.89	0.9614	Attention-based but single-dataset evaluation; no cross-domain validation	Used quaternion CNN features with channel–spatial attention
Sharma and Guleria (51)	VGG-16 with neural network classifier	Public pediatric CXR datasets	Normal vs. pneumonia	92.15–95.40	0.974–0.988	0.937–0.954	Performance varies across datasets; transfer learning model lacks uncertainty and calibration analysis	Included as a transfer learning benchmark for pediatric CXR pneumonia classification
Reshan et al. (53)	MobileNet-based transfer learning	Pediatric CXR dataset	Normal vs. pneumonia	94.23	0.972	0.9596	Lightweight model but limited explainability and limited cross-domain robustness testing	Included as a lightweight transfer learning benchmark for pediatric CXR comparison
An et al. [54]	EfficientNetB0–DenseNet121 attention ensemble CNN	Kaggle/Kermany pediatric CXR	Normal vs Pneumonia	95.19%	0.9564	0.9606	Single-dataset evaluation; no uncertainty estimation, calibration, or external validation	Combined EfficientNetB0 and DenseNet121 with multi-head and channel attention to improve pneumonia classification performance
Kundu et al. (56)	Ensemble of GoogLeNet, ResNet18, DenseNet121	Kermany pediatric CXR	Normal vs. pneumonia	98.81	0.9835	0.9879	Ensemble improves performance but increases model complexity; explainability and calibration are limited	Also evaluated RSNA separately, making it useful for cross-dataset comparison

**Table 11:** Comparative studies using the RSNA pneumonia detection challenge dataset.

Study	Model/type	Dataset	Classes	Acc. (%)	AUC	F1	Gaps/limitations	Interpretive remarks
Satish Chandra et al. (55)	ResNet50 deep features+NGBoost classifier	RSNA Pneumonia Detection Challenge	Normal vs. pneumonia	98.00	0.99	0.97	Strong feature classifier pipeline but no prospective clinical validation; limited explainability beyond feature analysis	Uses RSNA Stage-2 pneumonia dataset with 26,684 CXR images
Kundu et al. (56)	Ensemble of GoogLeNet, ResNet18, DenseNet121	RSNA Pneumonia Detection Challenge	Normal vs. pneumonia	86.85	0.8685	0.8695	Performance was substantially lower on RSNA than on Kermany, illustrating domain and dataset dependence	Same model achieved 98.81% on Kermany but 86.85% on RSNA
Yu et al. (57)	Deep CNN-based abnormality classification	RSNA Pneumonia Detection Challenge	Normal vs. lung opacity	94.71	0.9804	NR	Focused on normal/lung opacity classification; therefore, it is not fully equivalent to binary pneumonia-vs.-normal classification	Useful RSNA-based comparison, but class definition differs slightly
Cicėnas et al. (58)	Transfer learning CNNs; best MobileNetV2	RSNA Pneumonia Detection Challenge	Pneumonia vs. non-pneumonia	90.95	0.9783	0.9331	Used processed/balanced RSNA data; limited clinical external validation	MobileNetV2 achieved the highest accuracy, AUC, and F1 among tested models

**Table 12:** Comparative studies using NIH chestX-ray14/chestX-ray8 datasets.

Study	Model/type	Dataset	Classes	Acc. (%)	AUC	F1	Gaps/limitations	Interpretive remarks
Wang et al. (50)	Weakly supervised CNN benchmark	ChestX-ray8/NIH	Multi-label thoracic disease detection	NR	0.6333	NR	Weak labels derived from radiology reports; the multi-label task differs from binary pneumonia classification	Dataset benchmark; should be used mainly as NIH dataset reference
Rajpurkar et al. (37)	CheXNet/DenseNet121	ChestX-ray14	Pneumonia among 14 diseases	NR	0.7680	0.435	Evaluated pneumonia in a multi-label setting; results are not directly comparable with binary Kaggle/RSNA studies	CheXNet exceeded average radiologist F1 on annotated pneumonia test set
Reshan et al. (53)	MobileNet-based transfer learning	NIH ChestX-ray14	Normal vs. pneumonia/pneumonia-related classification	93.75	0.972	0.9596	Dataset-specific result with limited uncertainty, calibration, and prospective validation analysis	Provides an additional MobileNet-based comparison for NIH ChestX-ray14
Guendel et al. (59)	Location-aware DenseNet (DNet/DNetLoc)	ChestX-ray14/PLCO	Multi-label thoracic abnormality classification	NR	0.731 official; 0.765 non-official	NR	Patient-wise splitting and location-aware learning improve benchmarking fairness; binary accuracy and F1-score were not reported	Reported pneumonia AUROC improves over the original ChestX-ray14 baseline on comparable splits
Baltruschat et al. (60)	ResNet/DenseNet multi-label comparison	NIH ChestX-ray14	Multi-label thoracic disease classification	NR	Up to 0.714	NR	Shows strong dependence on split strategy, transfer learning, and metadata; pneumonia is reported mainly through AUROC	Its comparison lists pneumonia AUROC values across evaluated configurations

**Table 13:** Harmonized and cross-domain evaluation studies.

Study	Model/type	Dataset	Classes	Acc. (%)	AUC	F1	Gaps/limitations	Interpretive remarks
Yu et al. (57)	Deep CNN with external/pediatric evaluation	Adult CXR+RSNA+pediatric CXR	Normal/abnormal, normal/lung opacity, normal/pneumonia	94.64–94.71	0.9444 external; 0.9851 pediatric	0.9463	Tasks were not identical across datasets; therefore, this is not a direct harmonized binary pneumonia benchmark	Supports need for multi-domain validation.
Zech et al. (61)	DenseNet-121 CNN pneumonia-screening model	NIH ChestX-ray14 + Mount Sinai Hospital+Indiana University datasets	Pneumonia screening/radiographic findings consistent with pneumonia	0.732 internal; 0.238 external	0.931 internal; 0.815 external	NR	Accuracy was threshold-dependent and set at 95% sensitivity, so AUC is the more reliable comparison metric; external generalization decreased and models learned hospital-specific signals	Evaluated 158,323 CXR images from NIH, MSH, and IU; the jointly trained MSH + NIH model achieved internal AUC = 0.931 but dropped to external IU AUC = 0.815, highlighting domain shift.
Bassi and Attux (62)	U-Net segmentation+DenseNet201 classifier	Mixed COVID-19/normal/pneumonia CXR+external Hannover set	COVID-19 vs. normal vs. pneumonia	78.7% ext. + seg.	0.916 ext. + seg.	0.719	External validation showed reduced generalization compared with internal testing; segmentation improved robustness	Without segmentation, F1-score was 0.649 and mean accuracy was 74%.
HybridCATNet (proposed, 2025)	Hybrid CNN-attention transformer model	Harmonized pediatric+RSNA	Binary: pneumonia vs. normal	96.93%	0.9953	0.9693	Addresses key gaps through harmonized multi-source testing, uncertainty awareness, Grad-CAM, SHAP, calibration, and cross-domain evaluation. Remaining gap: prospective clinical validation	Proposed method; best interpreted within harmonized and cross-domain comparisons rather than as a direct single-dataset benchmark.

NR, not reported; ROC-AUC, receiver operating characteristic area under the curve; CXR, chest radiograph.

Despite these strengths, the present findings should be interpreted as evidence of technical validity and clinical promise rather than immediate deployment readiness. First, although the harmonized multi-institutional dataset was balanced and generalization-tested with a cross-domain performance drop of no more than 2.1%, it still cannot fully represent global population diversity, scanner variation, institutional workflows, or disease prevalence patterns. Second, all evaluations were retrospective; prospective multi-center reader studies are required to quantify workflow impact, radiologist trust calibration, and safety under real clinical conditions. Third, using a frozen U-Net lung segmentation module may lead to vulnerability to atypical anatomy or severe pathology, even though it was isolated from classifier optimization. Future work should therefore investigate segmentation-free anatomical encoding and continual learning strategies for site-specific adaptation.

Finally, the current binary pneumonia formulation limits the model's differential diagnostic scope. Extending HybridCATNet to multi-label classification across broader RSNA and NIH label spaces will be essential for evaluating its usefulness in routine chest radiograph interpretation, where pneumonia often overlaps with other thoracic abnormalities. Overall, the proposed model provides a rigorously benchmarked, interpretable, and uncertainty-aware foundation for future prospective validation and clinically translatable AI-assisted pneumonia screening.

## 6 Conclusion

This study introduces HybridCATNet, a hybrid CNN–Transformer framework for pneumonia detection from chest X-ray images, designed to improve diagnostic accuracy, robustness, interpretability, and retrospective reliability. The model combines nine-channel early fusion of raw grayscale, lung-masked, and edge-enhanced CXR representations with a CSCAM to capture both local radiographic patterns and global anatomical context. In 10-fold patient-wise stratified cross-validation on the harmonized dataset, HybridCATNet achieved strong and stable performance, with a mean accuracy of 96.93%, sensitivity of 98.13%, specificity of 95.72%, MCC of 0.9389, and AUPRC of 0.9954. Ablation experiments confirmed the contribution of each architectural component, showing that the Transformer branch, CSCAM, early multichannel fusion, and focal-loss-based optimization collectively improved classification performance and calibration. The balanced training strategy using authentic radiographs, rather than synthetic oversampling, improved convergence efficiency and supported stable optimization. Uncertainty-aware inference using Bayesian dropout further enhanced calibration and identified high-uncertainty cases, particularly those with ambiguous radiographic patterns. Cross-domain evaluation on NIH ChestX-ray14 and COVIDx CXR-3 showed accuracies above 94.8%, with limited performance degradation, indicating promising generalization under dataset shift. Explainability analysis using Grad-CAM and SHAP demonstrated strong alignment with radiologist-annotated regions, including high spatial overlap and strong attribution faithfulness. Robustness testing under noise, brightness, and rotation perturbations showed performance loss below 1.2%, while subgroup analysis indicated no statistically significant age- or sex-based performance differences. Overall, HybridCATNet demonstrates strong retrospective performance, calibrated uncertainty, and quantitatively supported interpretability, making it a promising research-stage foundation for future prospective validation in AI-assisted pneumonia screening.

## Data Availability

The original contributions presented in the study are included in the article/Supplementary Material; further inquiries can be directed to the corresponding authors.
